# Yeast vaccine production platform for human and animal infectious diseases

**DOI:** 10.3389/fimmu.2025.1697177

**Published:** 2025-11-19

**Authors:** Abel Ramos-Vega, Elizabeth Monreal-Escalante, Bernardo Bañuelos-Hernández, Miriam Angulo, Edgar Trujillo, Carlos Angulo

**Affiliations:** 1 Centro de Investigación en Ciencia Aplicada y Tecnología Avanzada (CICATA) Unidad Morelos del Instituto Politécnico Nacional (IPN), Xochitepec, Morelos, Mexico; 2 Immunology & Vaccinology Group and Laboratorio Nacional Consejo Nacional de Humanidades, Ciencias y Tecnología (CONAHCYT) de Generación de Vacunas Veterinarias y Servicios de Diagnóstico (LNC-GVD, Centro de Investigaciones Biológicas del Noroeste, La Paz, Mexico; 3 Secretaria de Ciencias, Humanidades, Tecnología e Innovación (SECIHTI)-Centro de Investigaciones Biológicas del Noroeste, La Paz, Mexico; 4 Facultad de Agronomía y Veterinaria, Universidad De La Salle Bajio, León Guanajuato, Mexico

**Keywords:** recombinant protein expression systems, biopharmaceuticals, eukaryotic cell factories, immune responses, human and animal health

## Abstract

Yeasts have contributed to human and animal health through functional antigen production for vaccine formulations. Some yeast-made vaccines have become a reality for humankind because they have reached commercialization (hepatitis B, HPV, and tick parasitosis). Many other vaccine prototypes are under preclinical and clinical evaluations, hoping for their usage soon. Currently, genomes, genetic modification techniques, and industrial vaccine manufacturing have been successfully developed for *Saccharomyces cerevisiae*, *Komagataella phaffii (formerly Pichia pastoris)*, and *Hansenula polymorpha*. Moreover, several yeast species are under research as prospects for vaccine production systems, such as *Kluyveromyces lactis, Yarrowia lipolytica, Schizosaccharomyces pombe, Saccharomyces boulardii*, and *Komagataella phaffii*. This review was mainly focused on commercial human and animal vaccines, describing and discussing genetic engineering tools, downstream antigen purification processes, GMP according to regulatory issues, and identifying challenges and future directions on the use of yeast as a vaccine production platform to fight against infectious diseases.

## Introduction

Infectious diseases continue to pose a major global health burden, affecting both humans and animals (1). Moreover, zoonotic pathogens are a significant concern due to their ability to cross species barriers and cause widespread outbreaks, as exemplified by the recent COVID-19 pandemic (2). In this sense, the use of antibiotics and vaccination remains the most effective strategy for controlling and preventing infectious [zoonotic] diseases. Nonetheless, the excessive and indiscriminate application of antibiotics leads to several problems, including antimicrobial resistance, environmental contamination, and disruption of the microbial diversity balance (3). In such scenarios, vaccines continue to be the most effective and safest strategy for controlling infectious diseases. Conventional vaccines are based on whole inactivated, attenuated pathogens or purified antigenic components from pathogens, such as subunit proteins or toxoids, all of which are rationally designed to elicit a protective immune response. These platforms have demonstrated substantial efficacy in decreasing disease incidence and mortality caused by infectious agents (4). However, conventional vaccines present several limitations, including safety risks associated with live or attenuated organisms, the need for highly trained personnel, cold-chain dependency, longer development timelines, and high production costs, among others (5). Consequently, novel vaccines are investigated to surpass or mitigate those challenges, which ideally should be cost-effective, suitable for alternative delivery routes, and tailored to resource-limited settings.

The arrival of recombinant DNA technology marked a significant turning point in vaccine development, enabling the precise design and production of specific antigens, for instance, without the need to cultivate or inactivate whole pathogens (6). This advancement has facilitated the development of recombinant protein-based vaccines using expression platforms such as bacteria, yeast, plants, mammalian cells, and insect cells, offering enhanced safety features compared to conventional approaches (7). Vaccines produced using recombinant expression platforms offer potential advantages, including reduced biosafety risks, the possibility of alternative routes of administration, lower production costs, and suitability for resource-limited settings (8). In this regard, yeasts have been used as a host system for recombinant protein production. In line with recombinant vaccine development, Valenzuela et al. (1982) reported for the first time the production of hepatitis B virus surface antigen in yeast, which was later evaluated in animal models (9, 10). To date, only yeast-based vaccines for humans against hepatitis B and human papillomavirus (HPV) have been approved, using *Saccharomyces cerevisiae* and *Hansenula polymorpha* as expression platforms (11, 12). In animals, certain commercial yeast-based vaccines targeting cattle ticks have been developed using *Komagataella phaffii* (formerly *Pichia pastoris*) as the expression system (13–15). Currently, yeasts offer practical advantages such as ease of genetic engineering, rapid growth, high biomass yield, and the absence of endotoxins. These features, combined with their capacity to express structurally complex recombinant proteins, make them highly suitable for large-scale vaccine production (16). This review outlines the current landscape of commercial vaccines produced in yeast. Although only a few yeast-based vaccines have been approved to date, they demonstrate the potential of yeast as a safe and versatile platform for producing recombinant vaccines. Reviewing these vaccines, their applications, and limitations provides valuable insight and highlights directions to improve their effectiveness, scalability, and applicability for future development in both human and animal health.

## Yeast as a vaccine production platform: genetic engineering tools

Successful yeast heterologous protein expression depends on having different and efficient genetic engineering tools for cell transformation. The first genetic yeast transformation through DNA recombinant technology was reported by Hinnen et al. and Beggs in 1978 (17, 18). The initial attempt consisted of producing spheroplasts (cells without a wall) to introduce foreign DNA. Later in 1983, Ito et al. standardized a genetic transformation protocol for whole intact cells, opening the path for an easier method (19). Other methods have been developed over the decades to improve efficient yeast transformation for recombinant protein production (20, 21). Improving protein yield has been an achievement through 1) the plasmid design and 2) the evaluation of different promoters, both constitutive and inducible, to manage gene expression (22).

Since the 90s, different plasmids have been developed to regulate the expression of foreign genes. Yeast vectors could be classified into two groups: plasmids that contain the yeast centromere sequence (CEN) and plasmids with a 2 μ origin of replication (23). Plasmids with CEN (centromere) sequences are mitotically stable yeast replicates, with only a single copy present per cell, whereas the 2µ plasmids are multicopy (about 20 per cell). Christianson et al. (24) developed four episomal plasmids with a high copy number based on endogenous sequences of yeast replication origin 2µ from *S. cerevisiae* and the bacterial plasmid pBluescript, making a shuttle vector (24). Plasmids for protein expression have been mainly developed for *S. cerevisiae*, *K. phaffii, Yarrowia lipolytica*, and emerging yeasts used to express recombinant proteins (25–28).

Mumberg et al. (29) developed a series of plasmids to clone genes under the control of constitutive promoters, allowing different levels of protein yield (29). The different promoters used encompassed weak (CYC1) and strong (TEF and GPD) promoters. Interestingly, Drew and Kim (27) designed an expression plasmid using an inducible GAL1 promoter, the recombinant protein of interest, and the GFP-octa-histidine sequence, which is a multi-copy plasmid integrating by homologous recombination in *S. cerevisiae* (23).

There are many plasmids based on these basic genetic elements. An extensive review of promoters and terminators for the expression of recombinant protein in non-common yeast, including *K. phaffii* and *Y. lipolytica*, has recently been reviewed (30). Given the industrial importance of *K. phaffii* and its potential for vaccine production, we provide a brief description of the commonly employed promoter. Representative constitutive promoters include GAP, fsLovA, cTRDL, and TEF1; while inducible promoters, which use methanol as the inducer, comprise SNT5, iTRDL, and AOX1. Among these, the methanol-inducible AOX1 promoter is the most widely used, enabling high-level expression of heterologous proteins (31). This system is advantageous for producing vaccines, as it can yield proteins constituting up to 30% of total cell protein (32, 33). While the methanol-inducible system is effective, concerns regarding methanol’s toxicity and flammability have led to the exploration of safer alternatives to induction methods. For instance, some orthologous promoters from yeasts can outperform the expression levels of AOX1 promoter (34).

## Downstream antigen purification and additional bioprocesses

Downstream processing of yeast-made antigens involves a series of purification techniques essential for recovering and refining bioproducts from fermentation broths. This process is critical for ensuring product purity and reducing production costs, particularly in the pharmaceutical industry. The key aspects of downstream yeast-made antigen purification and additional bioprocesses will be outlined.

The initial steps often involve removing microbial cells through centrifugation and filtration, which are crucial for clarifying the fermentation broth (35). Techniques such as protein A chromatography are effective for purifying antibodies and removing contaminants like β-glucan, which can pose immunogenic risks (36, 37). Other methods include ion exchange and affinity chromatography, which are tailored to the specific properties of the target biomolecule (37).

The liquid-liquid extraction method is commonly used to extract fermentation products, allowing for the separation of desired compounds from complex mixtures (38). The next step is the scale-up of the fermentation process, which involves optimizing the bioreactor (pH, dissolved oxygen, and medium composition). The fermentation processes used by biopharmaceutical manufacturers have demonstrated increased quantities of therapeutic proteins. However, this increase subsequently leads to capacity bottlenecks in the purification process (known as downstream processing) and is associated with high costs. Downstream processing comprises up to 80 percent of the total production costs (39, 40). Producers are increasingly recognizing the urgent need for improvement and have shifted their focus from enhancing the production process (upstream) to refining the downstream process (41, 42). This includes examining operating modes (batch, fed-batch, and continuous) and their impact on productivity. For instance, Martinez-Hernández et al. (43) developed a fed-batch bioprocess to produce a recombinant vaccine against *Entamoeba histolytica* in *K. phaffii* under operational conditions suitable for large-scale bioprocesses (43). Following the scale-up, the production of the recombinant protein reached 0.43 mg/mL, marking a 12-fold increase in production, despite the presence of methanol and oxygen-limited conditions. A maximum volumetric productivity of 3.75 mg/L h was achieved in a bioreactor, compared to 0.26 mg/L h attained in a shake flask. The next step involves assessing post-purification antigen stability, estimating the effects of freeze-thaw and lyophilization cycles, and analyzing protein integrity by SDS-PAGE and Western blot (44). Finally, in process validation, reproducibility must be verified in large-scale production batches (e.g., 10 L to 60 L) (45).

The comparison of purification yields and efficiencies between yeast and *E. coli* for recombinant antigen production reveals distinct edges and challenges associated with each host. Yeast, particularly species such as *S. cerevisiae* and *K. phaffii*, has shown promising results in producing functional recombinant proteins, while *E. coli* remains a widely used system due to its established protocols and high yields. Yeast can produce highly immunogenic proteins, as seen in the manufacture of vaccines against hepatitis B and HPV (46). Also, advances in understanding yeast metabolism have led to improved yields, especially for complex proteins (47). Companies employ various techniques to achieve effective purification in commercial yeast-based vaccines, focusing on optimizing processes that enhance both the purity and recovery of recombinant proteins. For instance, the purification of the SARS-CoV-2 receptor-binding domain utilized mixed-mode chromatography followed by hydrophobic interaction chromatography, achieving over 99% purity with a yield of approximately 21 mg/L (48). Tam et al. (49) have successfully applied a two-step purification strategy involving ion exchange chromatography (IEC) and size exclusion chromatography (SEC) to purify hepatitis B surface antigen, yielding a purity of 95.48% and a recovery rate of 78.07% (49). Additionally, the characteristics of yeast-based vaccines and the assembly of virus-like particles (VLPs) significantly influence purification efficiency. Particularly, *K. phaffii* is widely utilized for producing VLPs due to its ability to generate high levels of antigens like the Hepatitis B surface antigen (HBsAg) and poliovirus VLPs (50). However, the assembly process and the intracellular localization of these antigens can complicate the purification process.

On the other hand, *E. coli* can achieve considerable yields up to 23 mg of purified protein per liter of culture (51), and secretory production methods simplify the purification and improve protein folding crucial for functional proteins (52). Moreover, *E. coli* strains engineered for better disulfide bond formation can produce biologically active proteins more effectively than yeast in some cases (53). In summary, *E. coli* remains a robust choice for high-yield production and efficient purification, particularly for simpler proteins, while yeast offers advantages in producing complex proteins and vaccines. However, the choice of host ultimately depends on the specific requirements of the recombinant protein being produced. Finally, all processes must comply strictly with Good Manufacturing Practice (GMP) regulations for clinical trials, including long-term stability evaluations for regulatory approval. The GMP regulations are compulsory for ensuring the safety and efficacy of vaccines produced from yeast for clinical trials (54). These regulations encompass a range of processes, from initial production to quality control, guaranteeing that vaccines meet stringent safety standards before human administration (55).

## Good manufacturing practices for compliance with regulatory issues

One of the key advantages of employing yeast systems for vaccine production lies in their well-established use in the biopharmaceutical industry. For several decades, yeasts have served as hosts to produce recombinant vaccines and other biotherapeutics approved for human use (8, 10). This historical precedent facilitates regulatory approval and streamlines compliance with GMP, ensuring that medicinal products are consistently produced and controlled according to rigorous quality standards appropriate for their intended use. GMP compliance is critical throughout the vaccine development pipeline, including preclinical stages, where assessments of immunogenicity, toxicity, and allergenicity are conducted using carefully selected and justified *in vitro* and *in vivo* models (56). These evaluations form the foundation for subsequent clinical trials, which are essential for regulatory approval (57).

The standards of GMP demand precise characterization and traceability of production strains, underscoring the advantages of yeast-based systems over mammalian cells in recombinant protein manufacturing. Yeast cultures are inherently less susceptible to contamination by bacteria, fungi, mycoplasma, and viruses, a resilience largely attributed to their ability to proliferate in acidic environments that suppress the growth of many microbial contaminants (58, 59). Notably, yeast-based production also circumvents the risk of endotoxin contamination, a common concern in bacterial systems due to lipopolysaccharide residues (60). Furthermore, VLPs generated in yeast offer a compelling alternative to conventional vaccines derived from attenuated or inactivated viruses. In addition to the economic benefits of yeast cultivation, VLPs lack genetic material, thereby enhancing their safety profile—particularly for immunocompromised individuals (61).

In general terms for vaccine production, adherence to local regulatory frameworks is necessary to avoid delays or complications; however, the primary GMP guidelines are issued by major international agencies such as the European Medicines Agency (EMA), the U.S. Food and Drug Administration (FDA), and the World Health Organization (WHO). To further harmonize regulatory requirements and reduce redundancy in clinical and non-clinical studies, the International Council for Harmonisation of Technical Requirements for Pharmaceuticals for Human Use (ICH) was established (http://ich.org/). The ICH framework represents a significant advancement in aligning global standards, minimizing unnecessary duplication of human trials, and reducing animal use without compromising the integrity of safety and efficacy data. Within the GMP framework, the ICH Quality Guidelines are particularly relevant to vaccine development. These guidelines span 14 core areas, covering topics from stability testing (Q1A–Q1F) to analytical procedure development (Q14), offering a robust foundation for ensuring product quality and regulatory compliance (62). In the United States, the FDA regulates vaccine development and manufacturing under Subchapter F—Biologics, while GMP requirements for drug manufacturing, processing, packaging, and storage are detailed in Subchapter C—Drugs: General. Additional relevant provisions are found in Subchapters D—Drugs for Human Use and A—General (63). The EMA complements these regulations with its Good Pharmacovigilance Practices (GVP) guidelines, which encompass risk management plans, including detailed product profiles (e.g., vaccine composition, adjuvants, preservatives, and residuals), safety specifications, pharmacovigilance strategies, post-authorization efficacy studies, and risk minimization measures (64). Similarly, the WHO provides comprehensive guidance, such as the Guidelines on Clinical Evaluation of Vaccines (65). Regulatory aspects have been fundamental to improving the safety and efficacy of vaccines against infectious diseases. In this context, yeast-based systems offer several advantages for vaccine development: enhanced safety due to the absence of lipopolysaccharides (LPS), and their recombinant protein-based nature enables safe administration in immunocompromised individuals, positioning them as a preferable alternative to attenuated vaccines; scalability enabled by the use of low-cost culture media with minimal risk of contamination; and strong immunogenicity through the potential to produce VLPs-based vaccines.

## Human vaccines produced in yeast

To date, at least several recombinant vaccines expressed in yeast have been approved by the U.S. Food and Drug Administration (FDA), European Medicines Agency (EMA), and WHO for human use (see **Table 1** and **Supplementary Figure S1**). Leading manufacturers in this field include Merck & Co., Inc., GlaxoSmithKline Biologicals, Dynavax Technologies Corporation, and Sanofi Pasteur Inc. While the vaccines from these pharmaceutical companies are widely used across many countries, it is worth noting that China has developed a significant portfolio of domestically produced vaccines. These include several HPV vaccines manufactured using yeast-based expression systems—some of which have already been approved, such as the Shanghai Zerun vaccine (Walvax)—as well as others currently in various clinical trial stages (66, 67). Notably, two yeast-derived vaccines—Mosquirix™ (GlaxoSmithKline) and R21/Matrix-M™ (Oxford University, Novavax, Inc., and Serum Institute of India)—have been recommended by the World Health Organization for the prevention of malaria in children residing in endemic regions (68).

**Table 1:** Recombinant yeast-expressed vaccines approved by the FDA, EMA, and WHO for human use.

Vaccine (brand, company)	Yeast species	Target disease	Recombinant protein composition	Dosage & route	Immune response	Approving agency	Reference
VAXELIS (MSP Vaccine Company)	*Saccharomyces cerevisiae* (for HBsAg)	Diphtheria, Tetanus, Pertussis, Poliovirus, *Haemophilus influenzae* type b, Hepatitis B	10 µg HBsAg antigen with potassium aluminum sulfate	3 intramuscular doses at 2, 4, and 6 months of age	Antibodies	FDA	(157)
Twinrix (GlaxoSmithKline Biologicals)	*Saccharomyces cerevisiae*	Hepatitis A and B	20 µg HBsAg antigen with aluminum phosphate and aluminum hydroxide	3 intramuscular doses at 0, 1, and 6 months	Antibodies	FDA	(158)
Recombivax HB (Merck & Co., Inc.)	*Saccharomyces cerevisiae*	Hepatitis B	5 µg (pediatric) or 10 µg (adult) HBsAg antigen with amorphous aluminum hydroxyphosphate sulfate	3 intramuscular doses at 0, 1, and 6 months	Antibodies	FDA	(159)
ENGERIX-B (GlaxoSmithKline Biologicals)	*Saccharomyces cerevisiae*	Hepatitis B	10 µg (pediatric) or 20 µg (adult) HBsAg antigen with aluminum hydroxide	3 intramuscular doses at 0, 1, and 6 months (4 doses in adults)	Antibodies	FDA, EMA	(160)
HEPLISAV-B (Dynavax Technologies Corporation)	*Hansenula polymorpha*	Hepatitis B	20 µg HBsAg antigen with CpG 1018 adjuvant (TLR9 agonist)	2 intramuscular doses at 0 and 1 month	Antibodies	FDA	(161)
GARDASIL (Merck Sharp & Dohme LLC)	*Saccharomyces cerevisiae*	Human Papillomavirus (Types 6, 11, 16, 18)	20 µg HPV 6 L1, 40 µg HPV 11 L1, 40 µg HPV 16 L1, 20 µg HPV 18 L1 VLPs with amorphous aluminum hydroxyphosphate sulfate	3 intramuscular doses at 0, 1, and 6 months	Antibodies	FDA, EMA	(162)
GARDASIL 9 (Merck Sharp & Dohme LLC)	*Saccharomyces cerevisiae*	Human Papillomavirus (Types 6, 11, 16, 18, 31, 33, 45, 52, 58)	30 µg HPV 6 L1, 40 µg HPV 11 L1, 60 µg HPV 16 L1, 40 µg HPV 18 L1, 20 µg each of HPV 31, 33, 45, 52, 58 L1 VLPs with aluminum hydroxyphosphate sulfate	3 intramuscular doses at 0, 1, and 6 months	Antibodies	FDA, EMA	(163)
Hexyon (Sanofi Winthrop Industrie)	*Hansenula polymorpha* (for HBsAg)	Hepatitis B Diphtheria, tetanus, pertussis, poliomyelitis and *Haemophilus influenzae* type b	10 µg HBsAg antigen	2 intramuscular doses eight weeks apart	Antibodies	EMA	(164)
Fendrix (GlaxoSmithKline Biologicals)	*Saccharomyces cerevisiae*	Hepatitis B for patients with kidney failure	20 µg HBsAg antigen adjuvanted with AS04C (3-O-desacyl-4’-monophosphoryl lipid A (MPL))	4 intramuscular doses at 0, 1, 2, and 6 months	Antibodies	EMA	(165)
Hexacima (Sanofi Winthrop Industrie)	*Hansenula polymorpha* (for HBsAg)	Hepatitis B Diphtheria, tetanus, pertussis, poliomyelitis and *Haemophilus influenzae* type b	10 µg HBsAg antigen	2 intramuscular doses eight weeks apart	Antibodies	EMA	(166)
HBVAXPRO (Merck Sharp & Dohme B.V.)	*Saccharomyces cerevisiae*	Hepatitis B	5 µg HBsAg antigen	3 intramuscular doses at 0, 1, 6 months	Antibodies	EMA	(167)
Vaxelis (MCM Vaccine B.V.)	*Saccharomyces cerevisiae*	Diphtheria, tetanus, pertussis, hepatitis B, poliomyelitis, and *Haemophilus influenzae* type b	10 µg HBsAg antigen adsorbed on amorphous aluminium hydroxyphosphate sulfate	2 or 3 doses, with an interval of at least 1 month	Antibodies	EMA	(168)
Mosquirix™ (GlaxoSmithKline)	*Saccharomyces* *cerevisiae*	Malaria and hepatitis B vaccine	25 µg of Portion of *Plasmodium falciparum* circumsporozoite protein fused to hepatitis B surface antigen (RTS) adjuvanted with AS01_E_	3 doses at 0, 1 and 2 months, with booster at month 20	Antibodies	WHO and EMA (for its use outside of the European Union)	(169)
R21/Matrix-M™ (Oxford University, Novavax, Inc., and Serum Institute of India)	*Komagataella phaffii*	Malaria	25 μg of *P. falciparum* circumsporozoite protein fused to hepatitis B surface antigen adjuvanted with Matrix-M™	3 doses at 0, 1 and 2 months, with booster at month 20	Antibodies	WHO	(170)

*Saccharomyces cerevisiae* remains the preferred host for recombinant antigen expression; however, the hepatitis B vaccine HEPLISAV-B, Hexyon, and Hexacima are produced in *Hansenula polymorpha*, whereas the R21/Matrix-M™ vaccine is produced in *K. phaffii*. These yeasts are classified as Generally Recognized As Safe (GRAS), capable of growing in low-cost media and yielding high levels of recombinant protein (11, 12). Despite the use of similar genetic transformation tools, these yeasts differ in their regulatory protein expression elements (69, 70). Currently, yeast-expressed vaccines target hepatitis B, malaria and various types of HPV, all formulated as virus-like particles (VLPs). VLP-based strategies offer enhanced immunogenicity compared to non-assembled recombinant proteins (71). Despite more complex proteins requiring mammalian systems, such as glycoprotein E in the SHINGRIX vaccine (produced in Chinese hamster ovary cells), many yeast-derived recombinant vaccines without multiple post-translational modifications and capable of forming VLPs have demonstrated efficacy rates exceeding 95% (72). The main immune response involved in these vaccines is through the production of highly neutralizing antibodies, where the adjuvants play a critical role in the immunogenicity of yeast-based vaccines. Most adjuvanted formulations rely on aluminum salts (see **Table 1**). An exception is Dynavax’s HEPLISAV-B, which incorporates CpG 1018, a Toll-like receptor 9 (TLR9) agonist. Additional examples include malaria vaccines. Mosquirix™ employs the AS01E adjuvant, which consists of *Quillaja saponaria Molina* fraction 21 (QS-21) and 3-O-desacyl-4’-monophosphoryl lipid A (MPL). In contrast, R21/Matrix-M™ utilizes the Matrix-M™ adjuvant—a saponin-based formulation developed by Novavax AB (Uppsala, Sweden)—which elicits both Th1 and Th2 immune responses.

Notably, several yeast-derived vaccine candidates are currently undergoing evaluation (see **Table 2**). One example is the same malaria vaccine R21/Matrix-M™ expressed in *H. polymorpha*, forming VLPs, which is being evaluated in adult populations (ClinicalTrials.gov ID: NCT05252845). On the other hand, several vaccine prototypes have been evaluated in preclinical studies, including those targeting complex antigens such as the SARS-CoV-2 spike protein and influenza hemagglutinin. These proteins require specific glycosylation patterns, which have driven the development of innovative strategies to optimize yeast expression systems for industrial-scale production (73–75).

**Table 2:** Examples of recombinant yeast-expressed vaccines in clinical trials.

Manufacturer	Antigen and target disease	Yeast	Phase
CDIBP	L1 antigen from HPV 6,11,16,18 types	*Hansenula polymorpha*	NDA submited
CNBG	L1 antigen from HPV 6,11,16,18,31,33,45, 52,58,59,68 types	*Hansenula polymorpha*	Phase III
Stemirna Bovax	L1 antigen from HPV 6,11,16,18,31,33,45, 52,58 types	*Komagataella phaffii*	Phase III
Serum Institute of India Pvt. Ltd.	R21 antigen fused with hepatitis B Surface, against malaria	*Hansenula polymorpha*	Phase I
Jiangsu RecBio	L1 antigen from HPV 6,11,16,18,31,33, 45,52,58 types	*Hansenula polymorpha*	Phase I
Shanghai Zerun (Walvax)	L1 antigen from HPV 6,11,16,18,31,33, 45,52,58 types	*Komagataella phaffii*	Phase I

CDIBP, Chengdu Institute of Biological Products; CNBG, China National Biotec Group; NDA, New Drug Application.

## Animal vaccines produced in yeast

There are many recombinant yeast-based vaccines at preclinical levels, particularly useful in livestock, poultry, aquaculture, and companion animals, providing targeted protection against pathogenic viruses, bacteria, and parasites (see **Supplementary Figure S1**) (8, 76, 77). In birds, yeast-produced vaccines include those targeting fowl adenovirus (FAdV) (78) and parasites such as *Eimeria tenella* (79), where *S. cerevisiae*-expressed antigenic proteins serve as effective subunit vaccines. For fish, yeast-based vaccines expressing antigens from Cyprinid herpesvirus 2 and 3 have been developed to protect species such as Gibel carp (*Carassius auratus gibelio*), common carp (*Cyprinus carpio*) against these viral infections (80–82). In cattle, recombinant yeast vaccines expressing proteins from *Babesia bovis* (83) (and *Theileria parva* (84) are being investigated to reduce the impact of tick-borne diseases. For pigs, yeast-expressed vaccines against porcine circovirus type 2 (PCV2) and African swine fever virus (ASFV) have shown promising immune protection (85, 86). In pets, experimental yeast-based vaccines have demonstrated strong immunogenicity against hookworm*, Ancylostoma caninum*, and canine distemper virus (CDV) in dogs and feline infectious peritonitis virus (FIPV) in cats (75, 87, 88). However, in terms of commercial availability for veterinary purposes, there are just a few recombinant vaccines expressed in yeast, precisely in *K. phaffii*, and only against ticks (13, 15, 89–91). Although there are several commercial subunit recombinant veterinary vaccines on the market, most of them have been produced in *Escherichia coli* or baculovirus systems (92, 93). The *E. coli* expression system is the most widely used due to its simplicity, rapid growth, and low production cost. It allows high yields of recombinant proteins in a short time, making it ideal for large-scale vaccine manufacturing (94, 95). However, *E. coli* lacks the machinery for post-translational modifications such as glycosylation, which can limit its use for complex eukaryotic antigens (96). On the other hand, the baculovirus system offers a eukaryotic environment that enables proper protein folding, disulfide bond formation, and post-translational modifications, producing more structurally and functionally authentic antigens. This system is especially valuable for glycoproteins and complex multimeric proteins used in vaccines (97, 98). The baculovirus system is more expensive and slower than *E. coli* but often improves vaccine production and efficacy of immunologically relevant proteins (99). In contrast, the yeast system is a cheap platform that can perform functional post-translational modifications, and its disadvantage is over-glycosylation of proteins or the production of non-mammalian glycan patterns, which could affect vaccine immunogenicity (100, 101). Here, the recombinant yeast-based vaccines commercially available for animals will be reviewed by analyzing doses, efficacy, and immune responses.

Regarding commercial vaccines, the yeast-made vaccine Gavac^®^ (Heber Biotec S.A., Havana, Cuba) and TickGARD^®^ (Biotech Australia Pty. Ldt.) are composed of recombinant *Rhipicephalus microplus* Bm86 gut antigen expressed in *K. phaffii* that allows protection against cattle ticks (13, 14, 90, 102). These vaccines are administered in the neck in multiple doses in a recommended schedule of two initial doses on days 0 and 28, followed by a booster dose every 6 months (103). The effectiveness of Bm86-based vaccines like Gavac^®^ and TickGARD^®^ has been assessed through parameters such as tick survival, egg production, and fertility. Studies show variable efficacy (>50% in some cases), influenced by tick strain and cattle breed. Interestingly, vaccines reduced acaricide use by up to 87% and saved $23.4 per animal/year (15). However, efficacy varies due to tick genetic diversity, particularly polymorphisms in the Bm86 gene (104). Additionally, in 2018, Mexico developed Bovimune Ixovac^®^ using the “Media Joya” strain and expressed it in *K. phaffii* (105). It reduced tick infestations to <10 per animal and tick baths from 14 to 3 per year, with an 86% drop in tick fertility. In Colombia, Tick-Vac^®^, developed by LIMOR and marketed by TECNOQUÍMICAS, uses Bm86 from tick larvae. It follows a three-dose initial schedule with biannual boosters. Field trials showed 80% protection, with clinical studies reporting 64–96% efficacy and reduced parasite loads across various agroecosystems (15).

The immunological outcomes behind vaccination rely on antigen-presenting cells like dendritic cells and macrophages that process and present Bm86 via bovine leukocyte antigen. This activates naive T cells, which help activate B cells in germinal centers, leading to the formation of plasma and memory B cells (106). Plasma cells produce specific antibodies, while memory cells remain in lymphoid organs awaiting booster doses. Antibodies (IgM and IgG) circulate in the blood and are ingested by ticks during feeding. They bind to Bm86 in the tick gut and activate the classical complement pathway, causing enterocyte lysis and impairing digestion and overall tick health (107, 108). In general, yeast-made veterinary vaccines are an option to fight against animal diseases that remains to be fully exploited.

## Limitations and future directions

**Figure 1:**
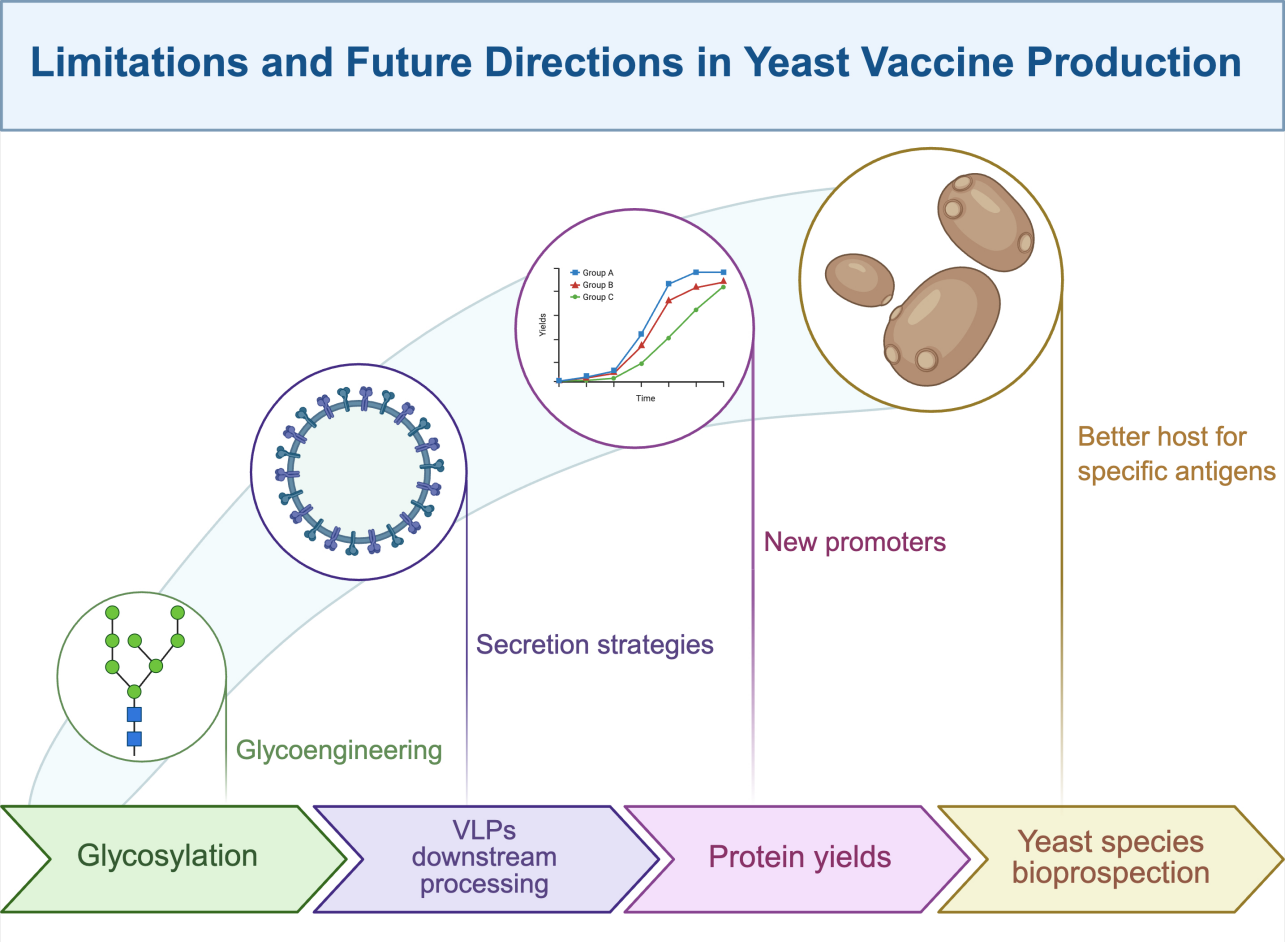
Limitations and future directions in yeast vaccine production.

Yeast as eukaryotic cells have many advantages to produce recombinant vaccines over mammalian cells and bacterial counterparts. However, several disadvantages have led to limited success in reaching commercial vaccines (See **Figure 1**). The main issues revolve around proper glycosylation, accurate formation of VLPs, and high yields. Although glycosylation is essential for the structural and functional integrity of many antigens—and yeast is capable of performing this post-translational modification—certain glycosylation patterns, such as high-mannose N-glycans, pose challenges to protein stability. These modifications can alter the expected glycosylation profile and critically impact both the immunogenicity and allergenicity of antigens (107). To address these issues, advances in glycoengineering have partially mitigated such limitations (109). In the context of yeast genetic engineering, efforts have focused on generating knockout strains of *S. cerevisiae* targeting genes involved in mannosylphosphate biosynthesis (110). Particularly, genes such as Mnn2p, Mnn11p, and α-1, 6-mannosyltransferase Och1p have been disrupted to reach this goal (29). Parallel strategies have explored the overexpression of endoglycosidase genes to enhance glycosylation efficiency (111). Both approaches hold promise for the production of complex antigens with more human-like glycosylation profiles.

An alternative strategy involves modifying antigen sequences to eliminate glycosylation sites. For instance, research groups developing yeast-based vaccines against SARS-CoV and SARS-CoV-2 have evaluated the expression in *K. phaffii* of deglycosylated receptor-binding domain (RBD) fragments. In these constructs, N-linked glycosylated asparagine residues were removed (112). The resulting proteins exhibited high yields without compromising antigenicity and elicited even stronger neutralizing antibody responses than their glycosylated antigen. Building on this approach, subsequent work during the SARS-CoV-2 pandemic targeted Asn331 and Cys538, again demonstrating robust induction of neutralizing antibodies (74). Interestingly, the CRISPR-Cas technology has emerged as a novel tool for genome modification applied in yeast, achieving successful glycoengineering (113). Collectively, these efforts underscore the potential of yeast as a cost-effective platform to produce complex antigens from pathogens of epidemic and pandemic relevance.

As previously discussed, licensed yeast-derived vaccines in humans predominantly rely on VLPs as antigenic platforms. Despite their success, several challenges remain in the production and optimization of VLP-based vaccines. These include efficient VLP assembly and secretion, the formation of enveloped and non-enveloped VLPs, and the tendency of VLPs to aggregate—often necessitating the use of anti-aggregation agents (76, 114). In most cases, VLPs expressed in yeast accumulate intracellularly, which poses significant limitations for large-scale production due to the need for cell lysis and complex downstream processing (115, 116). To circumvent these issues and reduce purification costs, secretion strategies have been explored, notably through the use of signal peptides such as the α-mating factor secretion signal (117, 118). However, unlike other eukaryotic systems capable of producing enveloped VLPs via budding from the plasma membrane (119), yeast lacks this capability under normal conditions. Enveloped VLP production in yeast has only been achieved through the generation of spheroplasts by enzymatic removal of the cell wall (120). A promising strategy involves the co-assembly of VLPs incorporating multiple antigens. These multilayered or mosaic VLPs may enhance vaccine efficacy or confer multivalent protection against diverse viral strains or species (121, 122).

A relevant issue is the improvement of recombinant protein yields. This aspect has claimed attention for research focused on strong AOX1 and GAP promoters (123–125), along with new promoters recently reported (126, 127). Additionally, it is well-known that yeast-preferred codons enhance recombinant antigen production (128). Of interest to improve yeast-made vaccines is that higher copy gene number through repeated sequences can also proportionally increase antigen transcript and recombinant protein (129). Even if high recombinant protein yields can be accurately reached, the downstream protein recovery and purification process faces additional challenges. To surpass this issue, for instance, cell disruption methods have been proposed for better recovery of recombinant antigens, especially those based on response surface methodology (130, 131). Innovations in protein purification have also been pursued, such as non-affinity chromatographic methods (132, 133). Moreover, improvements in classical ion (134, 135) and anion (136) exchange chromatography are being investigated to remove aggregates and optimize the purification process. Notably, current research on optimized buffers during down-processing is relevant for VLP-based recombinant vaccines (137). Lastly, partial purification as a downstream process using nano-colloidal silica adsorbent (Aerosil-380) can help in the separation of antigens (138), serving as a clarification process before final purification through non-affinity and affinity methods. In this regard, the use of nanoparticles is a future direction that promises high-efficiency nano-systems for the purification of antigens produced in yeast (139).

It is convenient to mention that the production of commercial recombinant vaccines in yeast has been limited to *S. cerevisiae, K. phaffii*, and *H. polymorpha*. However, many yeast species should be explored to find a better host to produce specific antigens on a case-by-case basis. It means a battery of available yeasts to select the best host in terms of yields, functionality, and safety. Prospects in this direction include the use of *Kluyveromyces lactis* (140–142), *Yarrowia lipolytica* (140), *Schizosaccharomyces pombe* (143–145), and *Saccharomyces boulardii* (146), in which antigens have been recombinantly produced. In addition to commercial vaccines, efforts in research & development have led to experimental vaccines with promising outcomes to prevent human and animal infectious diseases. Among many others, the most recent studies include experimental vaccines against rabies (147), herpesvirus hematopoietic necrosis (148, 149), Covid-19 (150), polio (151), bovine mastitis (152), tuberculosis (153), ETEC (154), human papillomavirus type 52 (155), and cancer (156).

## Conclusion

Yeasts are an antigen production platform for which genetic engineering tools, downstream processes, and industrial manufacturing have led to the benefit of vaccine commercialization to prevent hepatitis B, HPV, malaria, and tick parasitosis (*Rhipicephalus microplus*). In addition, efforts in R&D of experimental vaccines in preclinical and clinical studies hope to reach commercialization soon to fight against human and animal infectious diseases.

## Supplementary material

Supplementary Figure 1 Most representative vaccines produced in yeast.
