# Supplementary figures and images for: Yeast vaccine production platform for human and animal infectious diseases
Source: Front Immunol. 2025 Nov 19;16:1697177. doi: 10.3389/fimmu.2025.1697177 (PMC12672891; doi:10.3389/fimmu.2025.1697177)

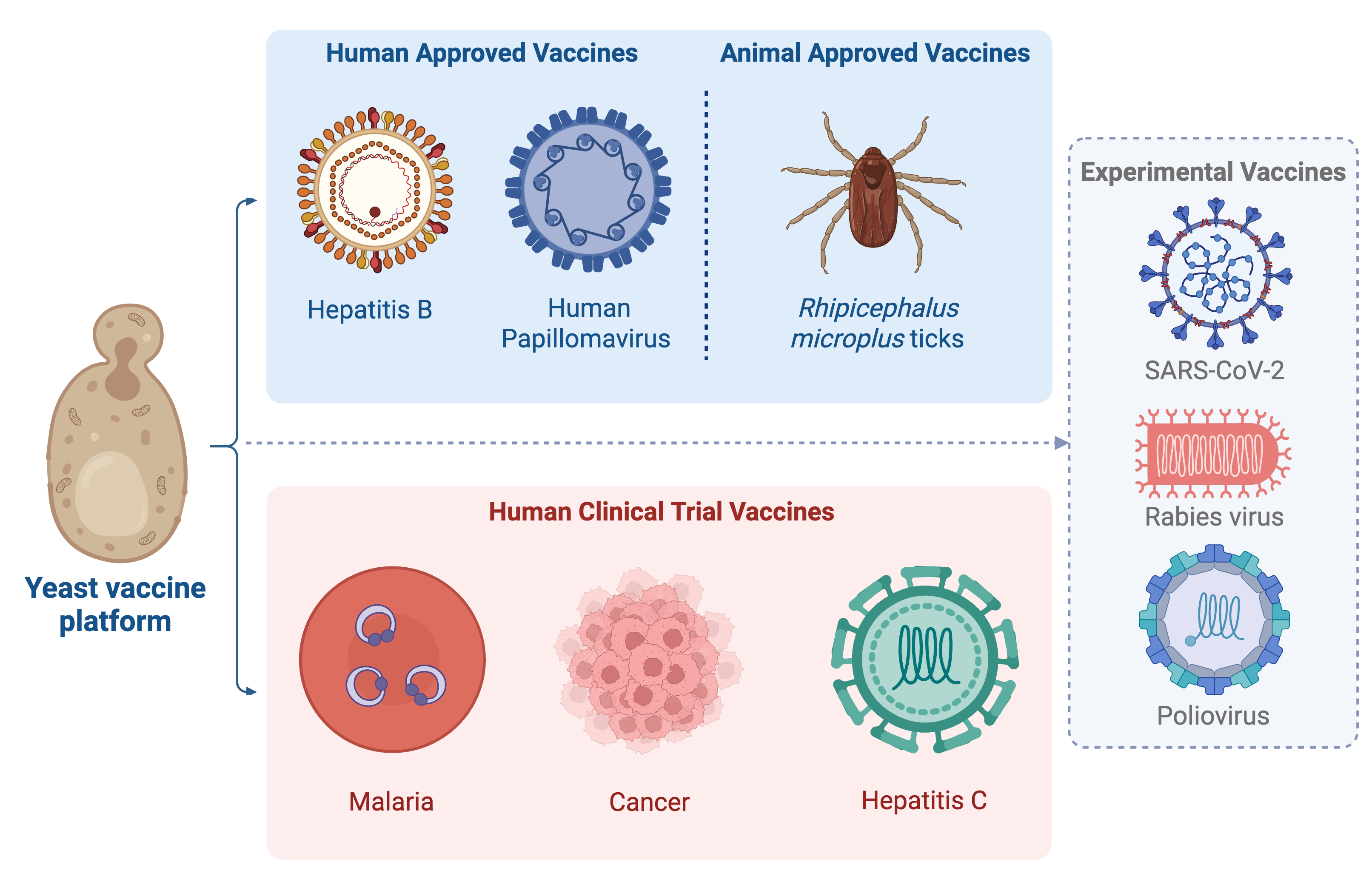

Supplement: Supplementary Figure 1 — Most representative vaccines produced in yeast. [file Image1.png]
